# Coupling coordination of the provision of medical services and high-quality economic development in the Yangtze River Economic Belt

**DOI:** 10.3389/fpubh.2023.1298875

**Published:** 2024-01-05

**Authors:** Shipeng Yang, Hongtao Yan, Yefang Gong, Siying Zeng

**Affiliations:** School of Public Administration, Xiangtan University, Xiangtan, China

**Keywords:** Yangtze River Economic Belt, provision of medical services, high-quality economic development, coupling coordination, panel Tobit model

## Abstract

**Background:**

Promoting high-level coupling coordination between the provision of medical services (PMS) and high-quality economic development (HED) has emerged as a critical issue in China’s pursuit of high-quality development and is now a significant subject of concern in the area of welfare economics.

**Materials and methods:**

Based on panel data from 11 provinces and municipalities in the Yangtze River Economic Belt, covering the period from 2010 to 2020, this study conducted an empirical analysis of the coupling coordination between PMS and HED and its influencing factors. Methods combined a comprehensive evaluation model, a coupling coordination model, and a panel Tobit model.

**Results:**

The study found that: (1) Regarding the overall situation in the Yangtze River Economic Belt, the overall PMS demonstrates a fluctuating upward trend, while HED fluctuates within the lower range of 0.3 to 0.4. The coupling coordination degree between PMS and HED fluctuates within the moderate range of 0.5 to 0.6. (2) In terms of the spatiotemporal evolution trends, there still exists substantial spatial disparity among provinces and municipalities within the Yangtze River Economic Belt regarding PMS; nonetheless, this gap is gradually narrowing. Significant regional disparities are also observed in HED, with Shanghai, Jiangsu, and Zhejiang leading among the provinces and municipalities in the Yangtze River Economic Belt. The coupling coordination degree between PMS and HED displays notable spatial discrepancies, where downstream areas of the Yangtze River Economic Belt such as Shanghai, Jiangsu, and Zhejiang exhibit a higher coupling coordination degree compared to other provinces and municipalities. However, most provinces and municipalities outside this group remain at a moderately coordinated stage concerning the degree of coupling coordination between PMS and HED. (3) Economic development level and local government competition had a significant negative impact on coupling coordination between PMS and HED, whereas there was a significantly positive impact on the degree of fiscal autonomy and urbanization.

**Discussion:**

This study contributes to comprehensively understanding the coupling and coordination relationship between the PMS and HED across provinces and municipalities in the Yangtze River Economic Belt. It provides empirical evidence for the collaborative evolution of PMS and HED.

## 1 Introduction

Ideally, the provision of medical services (PMS) and high-quality economic development (HED) should complement one another. However, is this truly the case in reality? According to the ‘Statistical Bulletin on National Economic and Social Development in 2022’ issued by the National Bureau of Statistics of China, the total gross domestic product (GDP) of China in 2022 was 121.0207 trillion yuan, an increase of 3% over the previous year at constant prices. Calculated at an annual average exchange rate, China’s total GDP was equivalent to approximately 18 trillion US dollars, ranking second worldwide. In 2022, China’s *per capita* GDP reached 85,698 yuan, representing a real increase of 3% from the previous year. At the annual average exchange rate, *per capita* GDP reached 12,741 US dollars, remaining above 12,000 US dollars for two consecutive years. However, under the Chinese-style fiscal decentralization system, local governments are keen to invest in economic growth and lack enthusiasm for investment in public services, such as medical and health services, which are heavily invested and slow to achieve results in the short term (1–3). To a certain extent, this has limited the provision level of medical services, and ‘difficult and expensive access to medical care’ remains a relatively prominent economic and social problem (4, 5). According to the 2019 Global Health Access and Quality (HAQ) Index released by the *Lancet*, China ranked only 48th. Regarding the efficiency of primary healthcare resource allocation in mainland China, existing research indicates that primary health care (PHC) institutions experienced significant technical inefficiency from 2012 to 2016, with less than 20% of provinces achieving technical efficiency every year (6). Simultaneously, there is a serious spatial imbalance in the supply of medical services in China; the Gini coefficient remains high, which is still far from the goal of “equalization” (7).

Regarding the relationship between medical services and the level of economic development, the formation of healthy human capital and the achievement of high-quality economic and societal development cannot be accomplished without guaranteeing high-level PMS (8, 9). Specifically, the development of basic medical and health services improves the health level of residents and favors the improvement of social labor efficiency and contribution, thereby playing a role in promoting economic growth (10, 11). Particularly after the COVID-19 pandemic, only countries with good medical infrastructure can hope to restart their economies rapidly (12, 13). Simultaneously, improvements in PMS should be based on a certain level of economic development. GDP growth increases government revenue, and an increase in government investment plays a decisive role in the development of public health undertakings (14). Thus, regardless of the expenditure preferences of local governments, a certain level of economic development is a mandatory for improving PMS.

Currently, there is limited research directly exploring the influencing factors of the coupling coordination degree between the provision of medical services and high-quality economic development. However, a literature review reveals that the level of economic development, local government competition, and fiscal autonomy exert varying degrees of influence on the interrelation between the provision of medical services and high-quality economic development. First, economic development can provide a material foundation for enhancing the quality of medical services. For instance, Cooper et al. found a significant correlation between the number of physicians *per capita* and the level of economic development (15). However, under a politically vertical evaluation mechanism, blindly pursuing economic development levels may potentially restrain high-quality economic development (16, 17). Hence, this study assumes that the level of economic development may exert a negative impact on the coupling coordination between the PMS and HED. Second, local government competition in China exhibits relatively unique institutional characteristics, manifested in the pursuit of ‘high-speed economic growth’ in practical scenarios (18, 19). Consequently, local governments have gradually transformed into ‘production-oriented governments,’ reflected in their expenditure structure that leans toward investing in productive public goods (20), thereby squeezing non-productive expenditures like the provision of medical services. Simultaneously, it is challenging for China to swiftly alter its long-standing pattern centered around GDP (19). Local government officials, aiming to secure political promotion through horizontal competition, might overlook the enhancement of long-term economic growth quality (21). Therefore, this study assumes that local government competition may negatively impact the coupling coordination between the PMS and HED. Finally, regarding fiscal autonomy, classical fiscal decentralization theories suggest that devolving expenditure responsibilities enables local governments, familiar with the needs of their jurisdiction’s residents, to provide better public services (22), thereby fostering local economic development (23). For instance, Sousa et al. found a positive correlation between local government fiscal autonomy and the efficiency improvement of medical services (24). Akai and Sakata’s research revealed a positive correlation between fiscal autonomy in U.S. states and local economic growth (25). Therefore, this study posits that fiscal autonomy may have a positive impact on the coupling coordination between the PMS and HED. Owing to the coupling coordination degree’s range falling between [0, 1], the conventional OLS model may yield biased and inconsistent parameter estimates. Since utilizing the Tobit model can address the issue of modeling restricted or truncated dependent variables (26). The academic community widely employs the Tobit model to study the influencing factors of coupling coordination degree (27, 28).

Economic development should enhance human well-being. China’s economy is currently in the process of shifting from a stage of rapid growth to one of high-quality development. High-quality development must be fundamentally guided by people’s aspirations and needs for a better life, with good health naturally serving as a fundamental foundation for a higher quality of life. PMS must adapt to people’s specific needs for medical services. The Chinese government must optimize PMS in the process of promoting HED, and vice versa. In this context, the relationship between PMS and HED is a crucial topic. In general, the abundant existing research has laid a solid foundation for further studies. However, the level of medical service provision and high-quality economic development in the Yangtze River Economic Belt has not been comprehensively measured, and the coupling coordination relationship between these two factors remains unclear. Moreover, there is a lack of exploration regarding the driving factors behind the coupling coordination of medical service provision and high-quality economic development. Therefore, based on panel data from 11 provinces and municipalities in the Yangtze River Economic Belt between 2010 and 2020, this study aims to quantitatively analyze the coupling coordination relationship between medical service provision and high-quality economic development, building on the evaluation index system for these aspects. It also explores temporal and spatial evolution patterns and driving forces. This study seeks to unveil the relationship between medical service provision and high-quality economic development in the Yangtze River region, aiming to facilitate the coupling coordination between the two and meet the increasing healthcare demands of the populace while promoting high-quality economic development.

## 2 Research design

### 2.1 Construction of indicator system

#### 2.1.1 Provision of medical services (PMS) system

Following the specific requirements of important documents such as ‘the outline of the 2030-year Plan of healthy China’ and ‘the National Health Plan of the 14th Five-year Plan’, and based on existing research (5, 29), an evaluation index system of medical and health service provision covering four dimensions and nine secondary indicators of human resources, financial investment, medical institution density, and institutional service capacity was constructed for this study (Table 1). First, healthcare services must be provided by professionals, which makes it a highly labor-intensive industry. If there is a shortage of professional human resources on the supply side of healthcare, the problem of ‘difficult access to healthcare’ is bound to arise. Second, when drawing fiscal budgets, local governments should allocate their limited financial resources to healthcare and other expenditures. The higher the proportion spent on healthcare, the greater the government’s support for this sector, and the more obvious the resulting output should be. Third, the density of healthcare institutions is an important indicator reflecting the spatial allocation of healthcare service resources, which is directly related to residents’ spatial accessibility to healthcare services. Finally, the institutional service capacity reflects the maximum extent to which regional healthcare institutions can provide healthcare services. Compared to selecting only a single input, output, or quality efficiency indicator, the results of an empirical evaluation obtained by constructing an evaluation index system for PMS are more comprehensive, and the reliability and validity are relatively higher. Compared to the existing index systems, the PMS evaluation index system constructed in this study incorporates relevant indicators of healthcare institution density, thereby enriching the multidimensional perspective of the evaluation index system. This enhancement aims to improve the scientific rigor and accuracy of measuring the level of medical service provision.

**Table 1:** Indicator system and influencing factors.

Target layer	Criterion layer	Indicator layer	Unit	Indicator attribute
PMS evaluation index system	Human resources	Number of health technicians per 1,000 technicians	Technicians/1,000 technicians	+
		Number of practicing (assistant) physicians per 1,000 physicians	Physicians /1,000 physicians	+
		Number of registered nurses per 1,000 nurses	Nurses/1,000 nurses	+
	Financial investment	Share of health expenditure in fiscal expenditure	%	+
		Financial expenditure on healthcare *per capita*	Yuan	+
	Medical institution density	Number of health institutions *per capita*	Institutions/10,000 institutions	+
		Number of health institutions per unit area	Institutions/10,000 km^2^	+
	Institutional service capacity	Number of beds in health institutions per 1,000 people	Beds/1,000 people	+
		Average number of beds in hospitals	Beds	+
HED evaluation index system	Innovation-driven development	Number of patents granted per 10,000 people	Patents/10,000 patents	+
		Net profit of high-tech enterprises as a proportion of GDP	%	+
	Coordinated development	Ratio of disposable income of urban and rural residents	%	−
		Ratio of added value of tertiary industry to added value of secondary industry	%	+
		Ratio of value added of secondary and tertiary industries to GDP	%	+
	Green development	Energy consumption per unit of GDP	Tonnes of standard coal/RMB 10,000	−
		Investment in pollution control as a proportion of GDP	%	+
	Development for global progress	Foreign investment as a proportion of GDP	%	+
		Total import and export of goods as a proportion of GDP	%	+
	Development for the benefit of all	Social security and employment expenditure/local fiscal expenditure	%	+
		Urban registered unemployment rate	%	−
Influencing factors	Economic development level (*pgdp*)	GDP *per capita*		
	Local government competition (*lgc*)	(Highest GDP *per capita* in neighboring provinces/GDP *per capita* in the province) x (Highest GDP *per capita* in the country/GDP *per capita* in the province)		
	Fiscal autonomy (*fa*)	General budget revenue/general budget expenditure		

#### 2.1.2 High-quality economic development (HED) system

Implementing new development concepts is the only way for China to achieve high-quality development and growth in the new era. In the process of HED, it is necessary to implement new development concepts fully, accurately, and comprehensively. Therefore, based on a deep understanding of the five major development concepts (i.e., innovation, coordination, green, openness, and sharing) and drawing on existing research experience (30, 31), this study constructed an evaluation index system of HED covering 11 secondary indicators from the five dimensions of: innovative development, coordinated development, green development, open development, and shared development (Table 1). There are significant differences in the geographical locations, resource endowments, and economic foundations of different regions in China. The process for achieving HED thus varies among regions, leading to significant differences in the characteristics and patterns of regional HED, which must be considered in the assessment. The chosen indicator system in this study was tailored to assess HED in the Yangtze River Economic Belt. This was evident in the selection of specific indicators, aligning with the requirements outlined in legal statutes, policies, and documents such as the Yangtze River Protection Law, the Development Outline for the Yangtze River Economic Belt, and the ‘14th Five-Year Plan’ for the Implementation of Development in the Yangtze River Economic Belt, all of which articulate relevant criteria for achieving high-quality economic development in the Yangtze River Economic Belt.

#### 2.1.3 Influencing factors

As previously mentioned, this study primarily explored the impact of the economic development level, local government competition, and fiscal autonomy on the coupling coordination relationship between PMS and HED. Among these factors, the level of economic development serves as the foundational support for the coupling coordination between the PMS and HED. Local government competition influences the policy orientation of local governments concerning PMS, HED, and related aspects, while fiscal autonomy reflects the capacity of local governments.

### 2.2 Model setting

#### 2.2.1 Comprehensive evaluation model

A comprehensive evaluation model was selected to measure the level of PMS and HED. First, the range method was used to perform dimensionless processing of the data for each indicator (32). The weight of each indicator was then determined using the entropy weighting method.

The comprehensive evaluation model (Eq. 1) is as follows:

(1) $$S=\overset{n}{\underset{i=1}{\sum }}\left(W_{j}\times Y_{k}\right)$$

where *S* denotes the composite index of health service provision or HED, *W_j_* denotes the weight of each indicator within the system, and *Y_k_* denotes the data for each indicator; *n* is the number of indicators, and *i* takes a value between [1, n].

#### 2.2.2 Coupling coordination model

In physics, coupling refers to the phenomenon in which two or more systems or elements influence one another through various interactions. The coupling-degree model can be used to calculate the interaction relationship between systems; however, it cannot easily reflect the level of coupling coordination. Therefore, the coupling coordination degree model was introduced to portray the coordination relationship between PMS and HED at each level of development and objectively reveal the level of coordination between PMS and HED.

The coupling model (Eq. 2) is shown as follows:

(2) $$C\left(S_{1}，S_{2}\right)=\frac{2\sqrt{S_{1}S_{2}}}{S_{1}+S_{2}}$$

where S_1_ and S_2_ denote the comprehensive indices of the healthcare service provision system and HED system, respectively. *C* denotes the coupling degree of the two systems, taking the value range of [0, 1], with a larger value of *C* indicating a stronger interaction between PMS and HED.

The coupling coordination model (Eq. 3) is as follows:

(3) $$D=\sqrt{C\times T\;},T=\alpha S_{1}+\beta S_{2}$$

where *D* denotes the coupling coordination degree, and *D*∈(0, 1); *C* denotes the coupling degree of the two systems; *T* denotes the comprehensive coordination index; *α* and *β* are the score weights of the healthcare service provision system and the HED system, respectively, and *α* + *β* = 1. This study considers that in the process of regional high-quality development, healthcare service provision and HED interact with each other and share an equal status. Therefore, the values of *α* and *β* were taken as 0.5, respectively.

To intuitively reflect the level of coupling coordination between healthcare service provision and economic quality development, drawing on existing practices (33–35), the coupling coordination degree was divided into five levels, as presented in Table 2.

**Table 2:** Classification of coupling coordination levels.

Coupling coordination interval values	Degree of coupling coordination	Coupling coordination level
[0.0, 0.2]	Extreme disorder	1
[0.2, 0.4]	Low coordination	2
[0.4, 0.6]	Moderate coordination	3
[0.6, 0.8]	High coordination	4
[0.8, 1.0]	Extreme coordination	5

#### 2.2.3 Panel Tobit model

Tobin (36) proposed an estimation method for limit-dependent variables using maximum likelihood estimation to solve the limit-dependent variable problem. As the value of the coupling coordination of the explained variables varies from 0 to 1, there is a characteristic of being cut, which is consistent with the setting conditions of the Tobit regression model for the limit-dependent variable. The panel Tobit models that are more mature in terms of development at this stage are the mixed Tobit model and the random-effects panel Tobit model. To obtain the best estimation results, both the mixed panel Tobit model and the random effects panel Tobit model were used, before finally the likelihood-ratio (LR) test was used to judge the existence of individual effects. If the model did not pass this test, a mixed Tobit model was required. To weaken the effect of heteroscedasticity, the level of economic development (*pgdp*) was logarithmised in the model. Based on the review and synthesis of existing studies, the following research hypotheses are proposed:

*Hypothesis 1*: The level of economic development exerts a negative impact on the coupling coordination between the PMS and HED.

*Hypothesis 2*: Local government competition has a negative impact on the coupling coordination between the PMS and HED.

*Hypothesis 3*: Fiscal autonomy has a positive impact on the coupling coordination between the PMS and HED.

The panel Tobit model (Eq. 4) is as follows:

(4) $$D_{it}=cons+\beta_{1}lnpgdp_{it}+\beta_{2}lgc_{it}+\beta_{3}fa_{it}+\varepsilon_{it}$$

where *D* denotes coupling coordination, *i* denotes region, *t* denotes time, *cons* is a constant term, *lnpgdp* denotes level of economic development, *lgc* denotes local government competition, *fa* denotes fiscal autonomy, and *ɛ* is a random disturbance term. *β*_1_, *β*_2_, and *β*_3_ are estimated coefficients for *lnpgdp*, *lgc*, and *fa*, respectively.

### 2.3 Study areas and data sources

**Figure 1:**
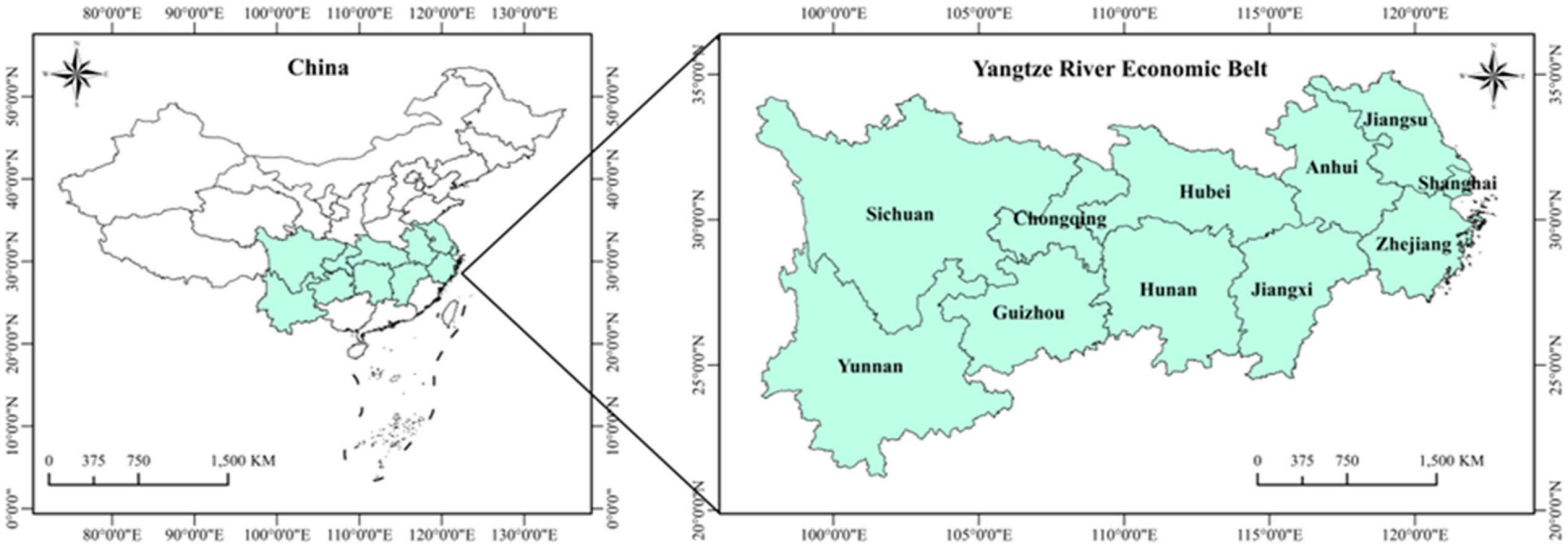
Schematic diagram of the study area.

The Yangtze River Economic Belt has become one of the regions with the strongest comprehensive strength and greatest strategic support in China, as well as the inland river basin economic belt with the largest development scale and widest impact area in the world. As Figure 1 shows, the Yangtze River Economic Belt spans three regions in East, West, and Central China, with a land area of approximately 2,052,300 square kilometers, and a population and GDP both exceeding 40.0% of the country as a whole. The Yangtze River Economic Belt is divided into upstream, middle, and downstream areas. The downstream area includes the four provinces and municipalities of Shanghai, Jiangsu, Zhejiang, and Anhui, and covers an area of approximately 350,300 square kilometers; the midstream area includes the three provinces of Jiangxi, Hubei, and Hunan and covers an area of approximately 564,600 square kilometers; and the upstream area includes the four provinces and municipalities of Chongqing, Sichuan, Guizhou, and Yunnan and covers an area of approximately 1,137,400 square kilometers. In 2022, the GDP of the Yangtze River Economic Belt reached 55.98 trillion yuan, accounting for 46.5% of the national total. Among them, six provinces—Jiangsu, Zhejiang, Sichuan, Hubei, Hunan, and Anhui—ranked among the top 10 in national GDP. The regional GDP of Jiangxi, Hunan, and Hubei increased by 4.7, 4.5, and 4.3%, respectively, ranking 1st, 3rd, and 6th in national growth rates. In 2022, the total import and export volume of the Yangtze River Economic Belt reached 19.30 trillion yuan, growing by 8.2% and accounting for 45.9% of the national total. In recent years, China has increased funding support for the health industry in the Yangtze River Economic Belt. From 2016 to 2018, a total of 293.74 billion yuan of medical and health subsidies were arranged by the National Health Commission for the Yangtze River Economic Belt. Additionally, the Ministry of Finance allocated 36.08 billion yuan of subsidies for health projects in the Yangtze River Economic Belt through central transfers in 2019. From 2016 to 2020, the country has allocated a cumulative 62.14 billion yuan of central budget investment to support a total of 3,532 medical and health infrastructure construction projects in the 11 provinces and municipalities of the Yangtze River Economic Belt.

Panel data from 2010 to 2020 across 11 provinces and municipalities in the Yangtze River Economic Belt were selected as the sample set. Data for the evaluation index system and influencing factors of high-quality economic development primarily originated from the ‘China Statistical Yearbook’ spanning 2011 to 2021. Data for the evaluation index system of medical and health service provision mainly came from the ‘China Health Statistics Yearbook’ covering 2011 to 2021. For certain indicators not available in the ‘China Statistical Yearbook’ or ‘China Health Statistics Yearbook,’ data were sourced from provincial statistical yearbooks and various economic and social development statistical bulletins of the respective provinces and municipalities. Interpolation methods were employed to address some missing data present in the research dataset.

## 3 Results

### 3.1 Trend analysis at the regional level

**Figure 2:**
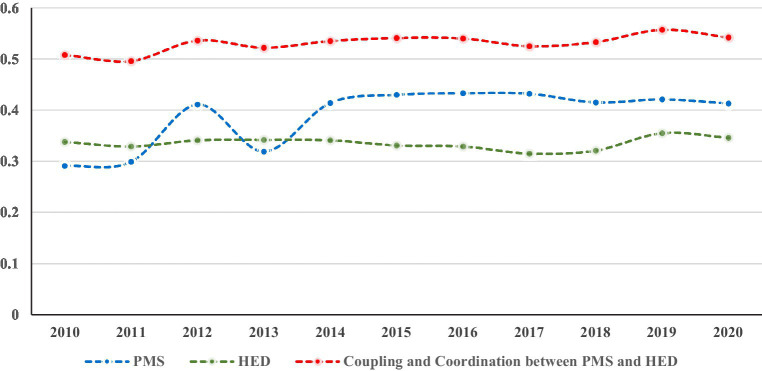
Evaluation of PMS, HED and their coupling coordination.

As shown in Figure 2, PMS improved significantly across the Yangtze River Economic Belt from 2010 to 2020, rising from 0.299 to 0.413. After experiencing a large degree of fluctuation between 2011 and 2014, it returned to a relatively stable state after 2015, before experiencing a slight downward trend after 2018. HED remained stable at a low level of 0.3–0.4, before a relatively large increase set in from 2019 onward. Whether this trend will continue remains to be determined. The coupling coordination between PMS and HED hovered at a moderate level of 0.5 to 0.6 from 2010 to 2020, exhibiting a slow rising state of fluctuation on the whole. Overall, PMS and HED remained in the moderate coupling coordination stage for a long time, and appeared to be far from a state of high coupling coordination.

### 3.2 Analysis of spatiotemporal change trend

#### 3.2.1 Spatiotemporal characteristics of PMS

**Figure 3:**
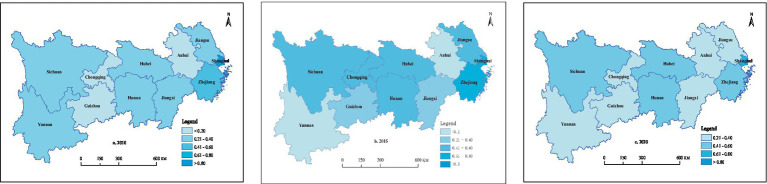
Spatial distribution of PMS in 2010, 2015, and 2020.

Cross-sectional data from 2010, 2015, and 2020 were selected to analyze the spatiotemporal distributional characteristics of PMS in the provinces and municipalities of the Yangtze River Economic Belt. In 2010, Shanghai (0.878) had a PMS value above 0.8, while none of the other provincial regions had a PMS value above 0.500. The PMS value of Anhui (0.156), Chongqing (0.170), and Guizhou (0.146) did not exceed 0.200; the PMS value of Jiangsu (0.229), Jiangxi (0.215), Hubei (0.279), Hunan (0.260), Sichuan (0.213), and Yunnan (0.211) did not exceed 0.300; and the PMS value of Zhejiang was 0.440, indicating that the PMS of all provinces and municipalities in the Yangtze River Economic Belt was at a relatively low level at that time, except for Shanghai. In 2015, within the Yangtze River Economic Belt, only three regions—Shanghai (0.759), Anhui (0.150), and Yunnan (0.194)—experienced a slight decline in PMS, while other areas witnessed varying degrees of improvement. Specifically, Zhejiang (0.645) and Hubei (0.576) had PMS values exceeding 0.500; Jiangsu (0.447), Hunan (0.449), Chongqing (0.445), and Sichuan (0.473) had PMS values surpassing 0.400; and Guizhou (0.357) and Jiangxi (0.447) had PMS values surpassing 0.300 and 0.200, respectively. Generally, a significant spatial disparity was observed in PMS among provinces and municipalities within the Yangtze River Economic Belt, highlighting a substantial regional imbalance in medical service provision. In 2020, despite the impact of COVID-19, no significant overall decline was observed in the PMS of the provinces and municipalities in the Yangtze River Economic Belt, and all provinces and municipalities had a PMS value above 0.200. Among them, the PMS in Shanghai (0.731) still ranked first in the Yangtze River Economic Belt, followed by Hubei (0.540), Zhejiang (0.418), Hunan (0.420), and Sichuan (0.413), all of which had a PMS value above 0.400. Jiangsu (0.339), Chongqing (0.385), Yunnan (0.363), and Guizhou (0.354) all had an PMS value above 0.300, while Anhui (0.298) and Jiangxi (0.284) both had an PMS value above 0.200. As shown in Figure 3, PMS varied significantly among the provinces and municipalities of the Yangtze River Economic Belt from 2010 to 2020. However, the disparities between regions have gradually shown a tendency to narrow. The inter-provincial/municipal extreme values of PMS in the Yangtze River Economic Belt were 0.732, 0.610, and 0.447 in 2010, 2015, and 2020, respectively. This trend reflects China’s relatively significant achievements in promoting equal access to basic medical and health services. In the lower reaches, Shanghai and Zhejiang consistently ranked among the top provinces and municipalities in terms of PMS; in the middle reaches, Hunan and Hubei had a relative advantage in PMS; and in the upstream region, Sichuan had a relative advantage over the other provinces and municipalities in this sub-region.

#### 3.2.2 Spatiotemporal characteristics of HED

**Figure 4:**
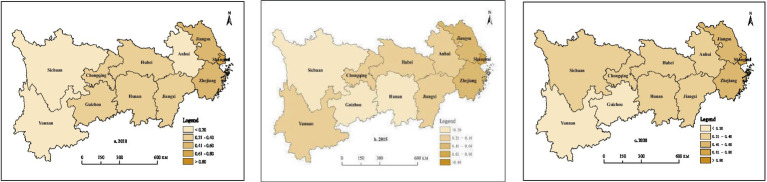
Spatial distribution of HED in 2010, 2015, and 2020.

Cross-sectional data from 2010, 2015, and 2020 were selected to analyze the spatial distribution characteristics of HED in the provinces and municipalities of the Yangtze River Economic Belt. From 2010 to 2020, only Shanghai, Jiangsu, and Zhejiang in the Yangtze River Economic Belt had HED values greater than 0.500. Among them, Shanghai’s HED increased from 0.775 to 0.803, Jiangsu’s HED decreased from 0.587 to 0.539, and Zhejiang’s HED increased from 0.527 to 0.532. From 2010 to 2020, HED showed an upward trend in five provinces and municipalities (Anhui, Jiangxi, Hunan, Chongqing, and Sichuan). Among them, HED increased from 0.183 to 0.314 in Anhui, from 0.239 to 0.257 in Jiangxi, from 0.206 to 0.220 in Hunan, from 0.264 to 0.301 in Chongqing, and from 0.170 to 0.282 in Sichuan. HED in Hubei, Yunnan, and Guizhou exhibited a downward trend, and it decreased from 0.316 to 0.269 in Hubei, from 0.204 to 0.174 in Yunnan, and from 0.244 to 0.119 in Guizhou. In general, significant regional differences were observed in the HED of the Yangtze River Economic Belt (Figure 4), with the HED in the eastern coastal provinces and municipalities being much higher than in other provinces and municipalities.

#### 3.2.3 Spatiotemporal characteristics of the coupling coordination between PMS and HED

**Figure 5:**
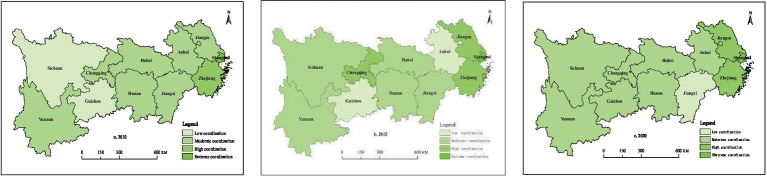
Spatial distribution of coupling coordination between PMS and HED in 2010, 2015, and 2020.

Cross-sectional data on the coupling coordination between PMS and HED in 2010, 2015, and 2020 were selected to conduct an in-depth analysis of the spatial distribution characteristics of the coupling coordination between PMS and HED concerning the coupling coordination stage divisions shown in Table 2. In 2010, the coupling coordination between PMS and HED was in an extremely coordinated stage in Shanghai; a highly coordinated stage in Zhejiang; a moderately coordinated stage in Jiangsu, Anhui, Jiangxi, Hubei, Hunan, Chongqing, and Yunnan; and a low-coordinated stage in Sichuan and Guizhou. In 2015, the coupling coordination between the PMS and HED among provinces and municipalities in the Yangtze River Economic Belt remained in a continuously dynamic phase, witnessing a growing number of regions reaching a highly coordinated stage. Shanghai’s coupling coordination between PMS and HED was at an extremely coordinated stage, leading among all provinces and municipalities in the Yangtze River Economic Belt. Jiangsu, Zhejiang, and Chongqing were at a highly coordinated stage, while Hunan, Hubei, Jiangxi, Sichuan, and Yunnan were at a moderately coordinated stage. Anhui and Guizhou were at a low-coordinated stage. In 2020, Shanghai remained the only province and municipality whose coupling coordination between PMS and HED was at an extremely coordinated stage, while the coupling coordination between PMS and HED in Jiangsu and Hubei dropped to a moderately coordinated stage, and Anhui rose to a moderately coordinated stage. Overall, as Figure 5 shows, as of 2020, the coupling coordination between PMS and HED of the provinces and municipalities in the Yangtze River Economic Belt remained at a moderate coupling stage, with the eastern coastal provinces and municipalities outperforming the other provinces and municipalities.

### 3.3 Analysis of driving factors

#### 3.3.1 Benchmark regression results

Before conducting the panel Probit regression, a variance inflation factor (VIF) test was initially employed to examine if multicollinearity exists among the explanatory variables. As indicated in Table 3, the VIF values for all explanatory variables were below 10. According to existing research conventions, this suggests the absence of multicollinearity issues among the variables (37).

**Table 3:** VIF test.

Variables	VIF	1/VIF
lnpgdp	3.27	0.306139
lgc	2.77	0.361490
fa	2.63	0.380753
Mean VIF	2.89	

The coupling coordination degree measured above was set as the explained variable. A mixed panel Tobit model and a random effects panel Tobit model regression analysis were then conducted on each explanatory variable using Stata 17.0 software. According to the LR test results, the original hypothesis of mixed effects could not be rejected; therefore, the results of the mixed-panel Tobit model were selected for the regression analysis. As Table 4 shows, explanatory variables were added step-by-step from Models 1–4, and the regression results of Model (4) were analyzed. The influence coefficient of the level of economic development (lngdp) on coupling coordination between PMS and HED was −0.4803 (*p* < 0.01), indicating that the level of economic development has a significant negative impact on coupling coordination between PMS and HED to a considerably high degree. The influence coefficient of local government competition (lgc) on coupling coordination between PMS and HED was −0.0234 (*p* < 0.01), indicating that the greater the intensity of local government competition, the greater the negative impact on coupling coordination between PMS and HED. The influence coefficient of fiscal autonomy (fa) on coupling coordination between PMS and HED was 0.0052 (*p* < 0.01), indicating that an improvement in fiscal autonomy contributes to an improvement in coupling coordination between PMS and HED. However, it also shows that the degree of influence of fiscal autonomy on coupling coordination between PMS and HED remains relatively weak.

**Table 4:** Tobit model results.

Variables	Model (1)	Model (2)	Model (3)
lnpgdp	−0.1382*** (0.0308)	−0.2917*** (0.0274)	−0.4018*** (0.0186)
lgc		−0.0348*** (0.0054)	−0.0238*** (0.0035)
fa			0.0059*** (0.0006)
_cons	2.0088*** (0.3227)	3.8186*** (0.3058)	4.6271*** (0.2181)
N	121	121	121

Robust standard errors in parentheses; ***indicate the significance of 0.01.

#### 3.3.2 Robustness test

To ensure the robustness and reliability of benchmark regression results, it is essential to conduct robustness tests on benchmark regression models. Using appropriate and different econometric methods, an analysis of the factors influencing coupling coordination between PMS and HED is conducted. If the obtained results are consistent with the benchmark regression results, it can be considered that the robustness of the benchmark regression results in this study is high. The generalized linear mixed model can simultaneously consider the effects of fixed and random effects on the differences in coupling coordination between PMS and HED across provinces and over time, addressing the potential issue of omitted variables. Therefore, drawing on existing research experience (38), the generalized linear mixed model is employed to regressively analyze the factors influencing coupling coordination between PMS and HED. The regression results are presented in Table 5.

**Table 5:** Regression results of generalized linear mixed model.

Variables	Model (4)	Model (5)	Model (6)
lnpgdp	−0.1382*** (0.0268)	−0.2917*** (0.0386)	−0.4018*** (0.0385)
lgc		−0.0348*** (0.0068)	−0.0238*** (0.0063)
fa			0.0059*** (0.0010)
_cons	2.0088*** (0.2870)	3.8186*** (0.4395)	4.6271*** (0.4084)
N	121	121	121

Standard errors in parentheses; ***indicate the significance of 0.01.

As shown in Table 5, the results obtained by estimating the factors influencing the coupling coordination between PMS and HED using the generalized linear mixed-effects model are consistent with the benchmark regression results, indicating the robustness and reliability of the benchmark regression findings.

## 4 Discussion

### 4.1 Spatiotemporal development trend of PMS in the Yangtze River Economic Belt

From 2010 to 2020, despite significant spatial differences in the level of PMS, there was still a significant overall improvement in PMS across the provinces and municipalities of the Yangtze River Economic Belt. In March 2009, the proposal of the ‘Opinions of the CPC Central Committee and the State Council on Deepening the healthcare System Reform’^1^ marked the official launch of the ‘New Medical Reform.’ The ‘New Medical Reform’ aims to address the widespread problem of ‘difficult and expensive access to healthcare’ among the Chinese public, implement the public welfare nature of medical and public health undertakings, and achieve the goal of all citizens having access to basic medical and health services (39). Over the past decade or so, the central government and health authorities at all levels have made multifaceted efforts to gradually increase investment in medical resources, and the overall capacity for PMS has been enhanced to some extent (40). The main reason for the large fluctuations in PMS levels from 2010 to 2014 may be that the entire healthcare system underwent a large-scale adjustment and adaptation process during the initial stage of the implementation of the ‘New Medical Reform.’ Spatial differences in terms of PMS in the Yangtze River Economic Belt must also be considered due to long-standing imbalances in the provision of healthcare services across different provinces and municipalities, as well as significant differences in the level of economic development.

### 4.2 Spatiotemporal development trend of HED in the Yangtze River Economic Belt

HED had been at a relatively low level for a long time before demonstrating a rebound trend after reaching its lowest point in 2017 and a significant increase in 2019. HED decreased once more in 2020, possibly owing to the impact of the COVID-19 pandemic. In addition, significant spatial differences in HED are also present throughout the Yangtze River Economic Belt. There are three possible explanations. First, for a considerable period, GDP, as a benchmark indicator of economic performance (41), has served as the main basis for assessing officials in organizational departments. When economic performance is the main factor influencing the promotion of local officials, they will mainly focus on GDP growth (42), while potentially neglecting the quality of economic development to a certain extent. Second, there is a prominent imbalance in terms of the level of high-quality development throughout the regional economy (43). This is further compounded by the large gap between the level of HED experienced in other provinces and municipalities and that seen in Shanghai, Zhejiang, and Jiangsu, thus affecting the overall level of HED in the Yangtze River Economic Belt. Finally, under the policy guidance of the central government, the focus of the local governments’ economic development at all levels has gradually shifted from high-speed to high-quality development. However, the transition between economic development and economic restructuring is not a simple undertaking and requires a rather long process. In addition, large differences in the economic structure and capacity of different provinces and municipalities have resulted in differences in the levels of HED.

### 4.3 Changing development trend of the coupling coordination between PMS and HED in the Yangtze River Economic Belt

The changing development trend of the coupling coordination between PMS and HED reflects the dynamic change process of the relationship between PMS and HED. Although the coupling coordination between PMS and HED remained in the moderate coupling coordination stage during the study period, the entire developmental process exhibited a fluctuating, slow upward trend. In particular, there were large increases in the two periods of 2011–2012 and 2018–2019. Significant spatial differences were also present in the coupling coordination between PMS and HED throughout the Yangtze River Economic Belt, as evidenced by the fact that the coupling coordination between PMS and HED in coastal provinces and municipalities was significantly higher than that of other provinces and municipalities.

### 4.4 Analysis of the driving factors of the coupling coordination between PMS and HED

(1) The level of economic development was confirmed to have a significant negative impact on the coupling coordination between PMS and HED. There are two possible explanations for this occurrence. First, the level of economic development can indeed lay a solid material foundation for PMS. Simultaneously, the gradual transition of political performance appraisals has also led to increasing concern from local governments regarding PMS. Nevertheless, it may be difficult for China to completely forego its GDP growth target in the short term. Second, there is an inconsistency between the level and quality of economic development. When the policy instrument of the relevant authorities is factor accumulation, economic growth targets are negatively correlated with the quality of economic development. Policy authorities cannot achieve both economic growth rate and economic development quality targets, with the measures taken to achieve the growth target eroding the quality of economic development (43). In summary, under certain conditions, the relationship between the level of economic development, PMS, and the level of HED is almost entirely different. (2) Local government competition, which is the act of competition among local governments to pursue higher economic performance under the incentives of performance appraisal and official promotion, was confirmed to have a significant negative impact on the coupling coordination between PMS and HED. Under the dual pressures of the ‘promotion race’ and the ‘economic race,’ local governments are keen to pursue the total amount of economic growth, gradually creating a ‘race to the top.’ The subsequent neglect of the quality of economic development and the intensification of competition among local governments inhibit the high-quality development of the regional economy (44). Simultaneously, local government competition leads to a preference for investment over people’s livelihoods in the structure and direction of fiscal spending, which means that the more distorted the local government spending structure is (45), the more detrimental it will be to the provision of healthcare services. In short, the greater the intensity of local government competition, the more detrimental it is to the provision of healthcare services and HED, which, in turn, negatively impacts the coupling coordination between PMS and HED. (3) Fiscal autonomy was found to have a significant positive impact on the coupling coordination between PMS and HED. There are two possible explanations for this. First, the increased fiscal autonomy of local governments helps facilitate PMS. When local governments have insufficient financial resources of their own and must rely on financial assistance from the central government to fulfill their responsibilities for authority expenditure, the effectiveness of local public goods provision is impaired (46, 47). Some studies have found that fiscal decentralization can empower local governments to meet the public preferences of residents in their jurisdictions in a timely manner (48), and that expanding the taxation power of local governments can improve local healthcare services by optimizing the structure of fiscal expenditure (49, 50). An appropriate degree of fiscal autonomy can also help promote HED. Some studies have found that when there is a serious vertical fiscal imbalance coupled with excessive fiscal pressure, local governments are likely to be unable to bear the pain of structural adjustment brought about by the promotion of HED. Under these conditions, local governments will resort to formalism, short-term investment, and other biased behaviors ‘to feign compliance’ in the implementation of central government policies, thus undermining the potential for HED and causing losses in the quality of economic development (51). However, when local governments have a reasonable degree of fiscal autonomy, the inhibitory effect of fiscal decentralization on the quality of economic growth can be alleviated to a certain extent (52). Therefore, an appropriate increase in fiscal autonomy can help promote PMS and drive HED, thereby contributing to a high level of coupling coordination between both policy objectives. However, it is important to note that the extent of this influence remains relatively weak in light of the current situation. (4) The level of urbanization was also found to have a significant positive effect on the coupling coordination between PMS and HED. There are two possible explanations for this. First, as urbanization is based on the provision of public services, the level of urban public services directly determines the ability of urban areas to absorb people to live and work (53, 54). Therefore, urbanization is manifested in the form of a gathering process of rural residents in urban areas; however, in essence, it is a process of coordinating urban and rural development and promoting the improvement of public services, including healthcare and education (55). Urban areas are important spatial carriers of industrial development, and urbanization can be achieved through various means. Urbanization exerts the foot voting function through the ‘spatial spillover effect’ of infrastructure, promoting geographical division of labor and specialization, and contributing to the upgrading of industrial structure. With rapid urbanization, cities retain intensive and high value-added industries through the ‘selection effect’, promoting industrial transition and upgrading. Simultaneously, urbanization also improves the level of human capital through the ‘agglomeration effect,’ accelerates the introduction and absorption of technology, and promotes the upgrading of industrial structures (56). In conclusion, urbanization has a significantly positive impact on the coupling coordination between PMS and HED by improving PMS and promoting HED.

This research provides a factual basis for improving the supply of medical services and promoting HED in the provinces and municipalities of the Yangtze River Economic Belt. However, this study also has some limitations. First, large changes in the caliber of statistical data limited the selection of indicators and the timeframe of the study. Second, as a preliminary attempt to study the degree of coupling coordination between the provision level of medical services and HED, this study only explored this relationship at the provincial level. Future studies can consider further refining the study’s parameters to empirically analyze the degree of coupling coordination between PMS and HED at the municipal and county levels. Finally, owing to space limitations, this study only analyzed the impact of three factors on the degree of coupling coordination between the provision level of medical services and HED. The impact of other factors will be further explored in the future.

## 5 Conclusion and policy implications

### 5.1 Conclusion

High-quality development is a fundamental requirement for China to implement its new development concepts, determine development ideas, formulate economic policies, and implement macro-control in current and future periods. High-quality development includes many aspects, among which HED is the key foundation and an essential prerequisite (57). Simultaneously, it should also be recognized that although China has made a historic leap in terms of economic strength, there are still many choke points and bottlenecks present in promoting high-quality development, one of which is PMS (58). Promoting high-level coupling coordination between PMS and HED is an important issue that must be addressed to ensure China’s continued high-quality development. To this end, based on the panel data of 11 provinces and municipalities in the Yangtze River Economic Belt from 2010 to 2020, this study used a comprehensive evaluation model, a coupling coordination model, and a panel Tobit model to conduct an empirical analysis of the coupling coordination degree between PMS and HED in the Yangtze River Economic Belt and to determine its influencing factors. The results show that: (1). Regarding the overall situation of the Yangtze River Economic Belt, the PMS displays a fluctuating upward trend, while HED fluctuates within a lower range of 0.3 to 0.4. The coupling coordination degree between PMS and HED fluctuates within a moderate range of 0.5 to 0.6. (2) In terms of spatiotemporal evolution, significant spatial disparities persist in the PMS among provinces and municipalities in the Yangtze River Economic Belt. Shanghai and Zhejiang maintain superior PMS compared to other provinces and municipalities in the Yangtze River Economic Belt. However, the inter-provincial/municipal extreme values of PMS are displaying a decreasing trend, indicating a gradual reduction in spatial disparities. (3) There are significant regional disparities in HED among provinces and municipalities within the Yangtze River Economic Belt. Shanghai, Jiangsu, and Zhejiang lead in HED compared to other provinces and municipalities in the region. Anhui, Jiangxi, Hunan, Chongqing, and Sichuan show an upward trend in HED, while Hubei, Yunnan, and Guizhou exhibit a downward trend. (4) The degree of coupling coordination between the PMS and HED within the Yangtze River Economic Belt demonstrates significant spatial disparities. The downstream areas of the Yangtze River Economic Belt, including Shanghai, Jiangsu, and Zhejiang, exhibit higher levels of coordination between PMS and HED compared to other provinces and municipalities, while the majority of the rest are at a moderately coordinated stage. (5) Both the level of economic development and local government competition have a significant negative impact on the coupling coordination degree between PMS and HED. This suggests that solely focusing on GDP growth and higher levels of local government competition are detrimental to the coordinated development of PMS and HED. Fiscal autonomy shows a significant positive relationship with the coupling coordination degree between PMS and HED. Enhancing fiscal autonomy is conducive to promoting the coordinated development of PMS and HED.

### 5.2 Policy implications

Based on the empirical results, to promote the improved coupling coordination of PMS and HED in the Yangtze River Economic Belt, it is necessary to continue to improve the level of PMS and accelerate the realization of HED in this area. Since the market mechanism alone cannot effectively address the problems of the provision of public and quasi-public goods, the problem of equity and accessibility in the allocation of medical health resources, the problem of the aggregate balance of medical health resources, and the problem of the structural balance of medical health resources (59), the government’s role must be given full priority in promoting the improvement of healthcare service provision. HED is a systemic project that cannot be achieved without the government’s institutional provisions and active promotion. Based on the findings of this study, the government can further promote high-level coupling coordination between PMS and HED in the following respects:

(1) Change the policy instruments of economic development. When the policy instrument of policy authorities is not factor accumulation but innovation-driven development, the quality of economic development is an increasing function of the economic growth goal, and the speed and quality of economic growth can be achieved simultaneously. On this basis, it can also provide a more solid material guarantee for PMS. First, the provinces and municipalities in the Yangtze River Economic Belt should focus on synergising policies focusing on high-quality regional economic development and continuously enhancing regional coordination regarding high-quality regional economic development. Second, they should also focus on establishing and improving the institutional mechanism for innovation-driven development; breaking the relevant institutional constraints on the flow of innovative elements, such as technology and talent; promoting the participation and collaboration of multiple parties, such as governments, enterprises, universities, and research institutions among regions; and promoting the synergy of innovative elements across regions. Third, efforts should be made to improve the funding management system to adapt to the characteristics of the long cycle and slow output of basic research, gradually increasing the proportion of fixed investments in basic research in the innovation field, and guiding innovation resources to adapt to the practical needs of China’s industrial restructuring and transition. Finally, formal systems such as laws and regulations should be improved further to maintain market order and protect property rights.

(2) Promote orderly competition among local governments. First, the transition from passive yardstick competition to active benchmarking management must be promoted. Benchmarking management originates from the management methods adopted by enterprises to enhance market competitiveness, which is a process of mutual imitation and learning among enterprises designed to achieve innovation. Introducing this approach in the public sector can help promote the transition from passive expenditure competition among local governments to active multidisciplinary learning. Second, in terms of top-level design, the central government scientifically guides and constantly regulates competition among local governments through sound incentive appraisals and other system designs, giving full priority to the positive effects of expenditure competition among local governments in optimizing PMS and promoting HED. Finally, the competition and cooperation mechanisms among local governments must be both established and improved upon. The status, purpose, role, and scope of cooperation and competition among local governments must be determined in the form of laws, ensuring that cooperation and competition among local governments have a legal basis. Simultaneously, local governments should establish a mechanism for dialogue and consultation based on equality and mutual trust, adhering to the principle of ‘mutual benefit and win-win’ to balance their interests.

(3) Local governments’ degree of financial autonomy must be improved. First, the reform of the division of financial affairs and expenditure responsibilities among the central and local governments should be further optimized, This can be accomplished by building a more scientific and effective mechanism for the allocation of transfer payment funds, optimizing the financial sharing proportion of local governments, reducing the vertical financial imbalance among central and local governments, and giving full priority to the resource allocation advantages of local governments in terms of fiscal policy. Second, local governments should be guided to establish expenditure systems that match fiscal autonomy with high-quality development to meet the needs of improving the provision level of medical services and promoting HED, thereby achieving high-level coupling coordination between PMS and HED. Finally, the supervision and inspection of key funds, key projects, and the implementation of various policies and systems should be stepped up to ensure that financial resources are not misappropriated, lost, or wasted, and that the efficiency of the allocation and effectiveness of utilization of financial resources is improved.

## Data availability statement

The original contributions presented in the study are included in the article/supplementary material, further inquiries can be directed to the corresponding author.

## Author contributions

SY: Conceptualization, Formal analysis, Funding acquisition, Investigation, Project administration, Supervision, Validation, Validation, Writing – original draft. HY: Conceptualization, Formal analysis, Funding acquisition, Investigation, Project administration, Supervision, Validation, Visualization, Writing – original draft. YG: Formal analysis, Methodology, Resources, Writing – review & editing. SZ: Data curation, Writing – review & editing.

## Footnotes

^1^http://www.gov.cn/jrzg/2009-04/06/content_1278721.htm
